# Effects of a family-based lifestyle intervention on co-physical activity and other health-related outcomes of fathers and their children: the ‘Run Daddy Run’ intervention

**DOI:** 10.1186/s12889-023-15191-z

**Published:** 2023-02-15

**Authors:** Julie Latomme, Philip J. Morgan, Sebastien Chastin, Ruben Brondeel, Greet Cardon

**Affiliations:** 1 Department of Movement and Sports Sciences, Ghent University, 9000 Ghent, Belgium; 2 PRCPAN (Priority Research Centre for Physical Activity and Nutrition), School of Education, University of Newcastle, 2308 Newcastle, Australia; 3 Department of Physiotherapy and Paramedicine, School of Health and Life Sciences, Glasgow Caledonian University, Scotland, UK

**Keywords:** Intervention, Co-physical activity, Physical activity, Fathers, Children, Families

## Abstract

**Background:**

Fathers are important in establishing healthy behaviors in their children, but are rarely engaged in lifestyle programs. Focusing on physical activity (PA) of both fathers and their children by engaging them together in PA (i.e. “co-PA”) is therefore a promising novel strategy for interventions. The study aim was to investigate the effect of the ‘Run Daddy Run’ on co-PA and PA of fathers and their children, and secondary outcomes such as weight status and sedentary behaviour (SB).

**Methods:**

This study is a non-randomized controlled trial (nRCT), including 98 fathers and one of their 6 to 8 years old children (intervention = 35, control = 63). The intervention was implemented over a 14-week period, and consisted of six (inter)active father-child sessions and an online component. Due to COVID-19, only 2/6 sessions could be implemented as planned, the remaining sessions were delivered online. In November 2019-January 2020 pre-test measurements took place, and post-test measurements in June 2020. Additional follow-up test was conducted in November 2020. PA (i.e. LPA, MPA, VPA and volume) of fathers and children were objectively measured using accelerometry, co-PA and the secondary outcomes were questioned using an online questionnaire.

**Results:**

Significant intervention effects were found for co-PA (+ 24 min./day in the intervention compared to the control group, p = 0.002), and MPA of the father (+ 17 min./day, p = 0.035). For children, a significant increase in LPA (+ 35 min./day, p < 0.001) was found. However, an inverse intervention effect was found for their MPA and VPA (-15 min./day, p = 0.005 and − 4 min./day, p = 0.002, respectively). Also decreases in fathers’ and children’s SB were found (-39 min./day, p = 0.022 and − 40 min./day, p = 0.003, respectively), but no changes in weight status, the father-child relationship, and the PA-family health climate (all p > 0.05).

**Conclusion:**

The Run Daddy Run intervention was able to improve co-PA, MPA of fathers and LPA of children, and decreasing their SB. Inverse intervention effects were however found for MPA and VPA of children. These results are unique given their magnitude and clinical relevance. Targeting fathers together with their children might be a novel and potential intervention strategy to improve overall physical activity levels, however, further efforts should however be made to target children’s MPA and VPA. Last, replicating these findings in a randomized controlled trial (RCT) is recommended for future research.

**Trial registration number:**

This study is registered as a clinical trial (clinicaltrials.gov, ID number: NCT04590755, date: 19/10/2020).

## Background

Physical inactivity is a global pandemic and a leading cause of physical and mental health issues [1]. A physically inactive lifestyle develops early in life and tends to track through life [2]. Yet, in Europe, up to 17% of primary school-aged children do not meet the physical activity guideline of on average 60 min of moderate-to-vigorous physical activity (PA) per day, across the week [3]. Therefore, PA promotion in children has become a research priority in public health [4].

Lifestyle interventions targeting children’s health behaviours, including PA, have often included parents as an important focus of change, as parents play a critical role in the health behaviours of their children [5, 6]. However, recent research has indicated that family-based interventions mainly include mothers, while fathers have been largely underrepresented [7, 8]. Fathers have rarely been targeted exclusively, and their influence on their children’s health and health behaviours is commonly overlooked [9–12]. This is an important gap, as recent research has indicated that fathers play a key role in the development of behaviors in their children [13–15], and especially their PA [12, 16, 17]. For example, one of our recent studies has shown that the association between fathers’ and children’s weight status is (partially) mediated through their PA levels [18]. This suggests that acting on PA of both the father and the child, that is, the father and the child being active together (“co-PA”) can be a novel and potentially effective intervention strategy [19]. In the present study, co-PA is defined as a form PA that includes “active play” which is physical, vigorous and highly stimulating and is jointly performed by the father and his child (e.g. playing soccer together, rough-and-tumble play, cycling) [20]. Besides its positive influence on the total PA levels of both fathers and children, co-PA may also influence the father-child relationship and the wellbeing of the child (i.e. self-esteem, self-regulation skills) [20–22]. To our knowledge, the effect of co-PA has only been tested in two Australian programmes (i.e. Healthy Dads Healthy Kids [13, 23] and DADEE (Dads and Daughters Exercised and Empowered) [24, 25]). Both programmes showed promising effects, with improvements in both fathers’ and children’s weight and PA, in the quality of the relationship between father and child, and the child’s social-emotional well-being. Despite its promising effects, the amount of experimental research investigating the effects of co-PA on the health behaviours (i.e. PA) and other health-related outcomes of both fathers and their children (i.e. SB, weight status, quality of the father-child relationship) is still scarce. This is especially the case in a European context, where to our knowledge, no such studies have been conducted.

It is highly recommended, when developing and evaluating an effective lifestyle intervention, that a theoretical framework is used, as clear theoretical underpinnings are known to maximize the potential for intervention effectiveness [26, 27]. Therefore, The Behavior Change Wheel was used as a theoretical framework to systematically develop the intervention [28, 29], which is described into detail in the Study Protocol paper of this study [30]. In brief, the intervention development followed three stages: (1) understanding the health problem and specifying the target behavior(s), (2) translating the findings into an intervention, and (3) refining and pre-testing the intervention [33]. Moreover, it is also important to take the perspective of the end-users, fathers in this case, into account when developing an intervention as most of the existing lifestyle interventions do not meet fathers’ needs and preferences [31]. A co-creation approach is often used in health promotion intervention studies to target this issue [32–34], which is thought to be an approach to increase adherence and enhance effectiveness [35–37]. Combining a theoretically based approach with a co-creation approach is innovative in this research field, leading to the further development and improvement of effective lifestyle interventions for fathers and their children across the globe.

The primary goal of the present study was to evaluate the efficacy of a newly developed lifestyle intervention for fathers and their children (i.e. ‘Run Daddy Run’ intervention) [30], on objectively measured PA of fathers and their children (6–8 years old), by increasing co-PA. We hypothesized that in the intervention group (compared to the control group), father-child co-PA and fathers’ and children’s PA will significantly improve.

## Methods

### Study design

**Fig. 1:**
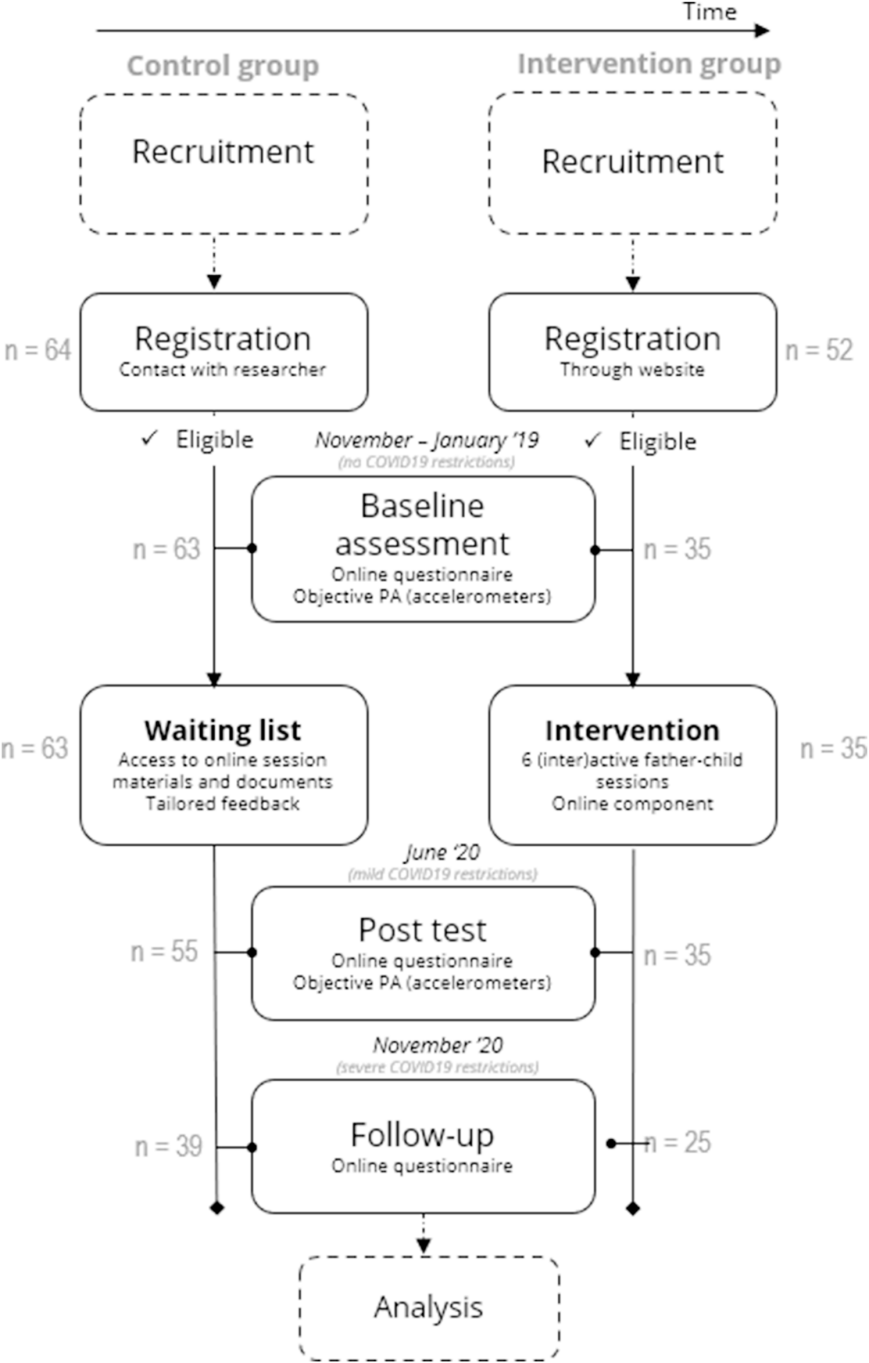
Study design and participant flow throughout the trial

The design of the Run Daddy Run study was a non-randomized controlled trial (nRCT), with a two-group pretest-posttest control group. In this design, participants were recruited in sequence (see Fig. 1 for the study design and participant flow) through convenience and snowball sampling (**see Participants and Recruitment** for more information on these recruitment strategies). This was done because the demand of study participation would differ significantly between the control group and intervention group. After the entire control group was recruited, participants for the intervention group were recruited, who were informed that they would participate in a programme promoting PA in fathers and children through 6 interactive sessions including an online eHealth component (see section **the Run Daddy Run intervention** for more information on the intervention). Participants of the control group were asked to participate in the pre-, post-, and follow-up measurements. Furthermore, the control group was told that they would receive a written report on their personal data collected at pre-test (e.g. PA levels, BMI, etc.) and that they would get access to the intervention materials after the Run Daddy Run intervention took place (see section **the Run Daddy Run intervention** for more information on the intervention materials).

Measurements were performed immediately before the intervention (baseline) and immediately after the 14-week intervention (post-test), in June 2020. Five months after the intervention, follow-up measurements were conducted which was one year after the baseline measurements (November 2020). Follow-up measurements were conducted to investigate whether or not intervention effects would be sustained over a longer time period. For the timing of these measurements, see Fig. 1.

### The Run Daddy Run intervention

#### Development of the intervention

The Behaviour Change Wheel was used as a theoretical framework to systematically develop the intervention [28, 29]. The Behavior Change Wheel is a comprehensive framework for systematically developing complex behavior change interventions, identifying important sources and interactions of behaviors to understand and target mechanisms of action within an intervention [29]. Details on the intervention development process can be found in Study Protocol of this paper [30], but briefly, three stages were followed using the “Guide to using the behaviour change wheel” [29]: (1) understanding the behaviour, (2) translating findings into an intervention, and (3) refining and pre-testing the intervention. Additionally, a co-creation approach was used for the intervention development process [34]. An overview of how this co-creation approach was combined with the Behaviour Change Wheel can be found in the Study Protocol of this study, where each step is described into detail [30].

#### Content of the intervention

The aim of the intervention was to improve father-child co-PA, and consequently the objectively measured PA levels of both. Secondary aims of the intervention were to target (co-) sedentary behaviour (SB), body mass index (BMI), family health climate regarding PA and the quality of father-child relationship. The Behaviour Change Wheel (BCW) was used as a theoretical framework to systematically develop the intervention [28, 29] together with a co-creation approach [30, 34]. In this approach, several co-creation sessions with fathers were organized to meet the needs and preferences of fathers, each covering a certain step of the Behaviour Change Wheel. Details on the intervention development (including the co-creation approach), and the specific intervention content can be found in the Study Protocol [30].

**Fig. 2:**
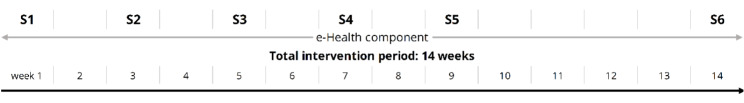
Timeline of the Run Daddy Run intervention under normal circumstances

In brief, the intervention was implemented over 14 weeks, including two components: (1) a practical component in which six (inter)active sessions were given face-to-face to the fathers and children, of 120 min each, and (2) an eHealth component which was implemented throughout the entire 14-week intervention period (for a timeline, see Fig. 2) [30]. The interactive sessions were delivered on a bi-weekly basis, except for the final session which was delivered four weeks after the preceding session, serving as a follow-up session (see Fig. 2). The interactive sessions were delivered by three facilitators: one main facilitator and two supporting facilitators, who were trained experts (i.e. Master students or graduated in movement and sports and/or health promotion sciences, educated for a full day to deliver the Run Daddy Run intervention).

**Fig. 3:**
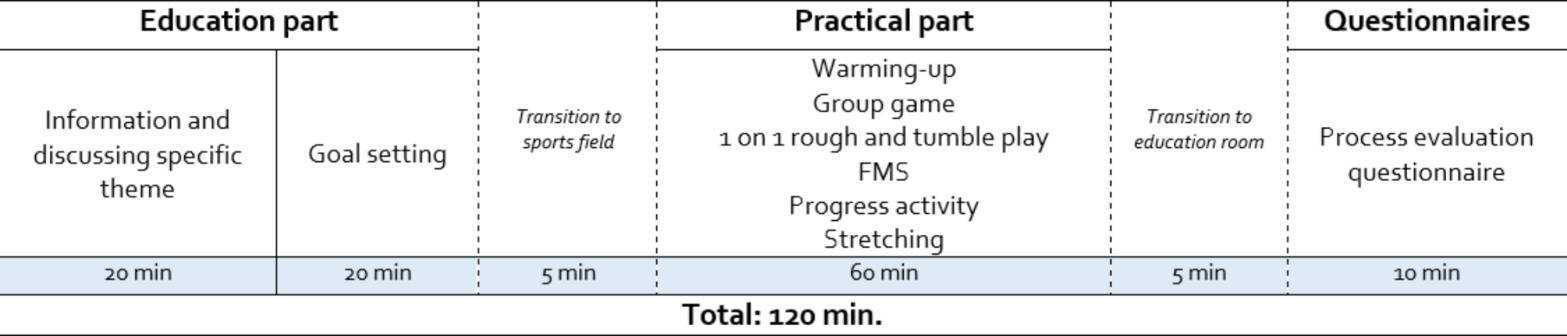
Timeline of one practical father-child session

For the intervention, participants were randomly assigned to a group, each containing about 12 father-child days. These groups received the same sessions, on a different evening in the same week. The main facilitator was always the same for the three groups, the supporting 2 facilitators were different for the three different groups, minimizing the change of a clustering effect due to participating in the same groups for the entire study duration. The face-to-face sessions took place at the same elementary school in Ghent, East-Flanders (Belgium). Each face-to-face session consisted of a 40-minutes education component and a 60-minute practical component part (see Fig. 3). In the education component, the lead facilitator educated the fathers and children on a key theme that fathers deemed important during the intervention development (see Table 1). More specifically, information on that specific theme was provided by the facilitator which was subsequently followed by a group discussion in which fathers could share their experiences on that topic.

**Table 1:** Overview of the Run Daddy Run themes across the six sessions

Session number	Theme	
	Education component *Theme based on identified barriers for co-PA according to fathers*	Practical component *Theme based on 2 to 4 FMS*
1	General introduction, importance of (co-)PA and role of the father	Jumping, landing, running and coordination
2	Motivation for (co-)PA *(i.e. information on different types of motivation and practical tips, which was based on the self-determination theory)*	Throwing, kicking, catching and rolling
3	Co-PA preferences and common co-PA interests	Rotating, rolling, pulling and pushing
4	Social support *(i.e. defining the concept how to receive and provide social support for co-PA, practical tips)*	Dribbling and striking
5	Sedentary behavior (screen time) *(i.e. defining the concept, importance of limiting this behavior, practical tips)*	Carrying, wheedling, crawling and lifting
6	Habit formation of PA and summary of all sessions	All FMS

After the education component, goals were set by the fathers and children on co-PA. In the subsequent practical component, several exercises were performed with each session focusing on specific fundamental movement skills (FMS) (see Table 1). The structure and approach in the practical component was modelled from the previous father-child co-PA interventions from Australia [13, 38] For the timeline of one such session, see Fig. 3.

Second, the eHealth component consisted of a website (www.rundaddyrun.be) where all intervention participants had access to a profile with a personal login and password. On this website, fathers and children were asked to set a weekly co-PA goal and log their co-physical activities performed at home (i.e. self-monitoring; a BCT selected in step 6 and 7 of the intervention development process, see [30]) in order to track progress and see if their goal was reached. Furthermore, on this website fathers and children had access to a large variety of physical activities and exercise ideas that could be performed together, with detailed instructions on how to perform them. Additionally, all intervention materials used in the interactive sessions could be found on this website (i.e. all information provided and documents used in the educational part of the sessions, (instructions on) all exercises performed in the practical part, etc.). Last, the eHealth component included a private chat group with the fathers and the facilitators, with the main goal to create a positive group atmosphere and group dynamics, and for the facilitators to send reminders. For more details on the eHealth component, see elsewhere [30].

### The intervention and restrictions during COVID-19

During the baseline measurements (November-January 2019–2020), no COVID-19 restrictions were applicable. During the post-test measurements (in June 2020), “mild” COVID-19 restrictions were applicable, meaning that (1) catering industry (e.g. restaurants, bars, cafés) and (non-essential) shops were open, (2) school were open (3) non-contact sports activities and facilities (both indoor and outdoor, amateur and professional) were allowed (4) social contacts were restricted to a maximum of 10 different people per week, and (5) teleworking was recommended but not mandatory. During follow-up measurements (i.e. November 2020),“strict” and strengthened COVID-19 restrictions were applicable [39], meaning that (1) catering industry and (non-essential) shops were closed, (2) schools were closed with (online) distance education, (3) outdoor exercise was allowed with a maximum of 4 people and sports clubs, swimming pools and fitness centers were closed (4) social contacts were restricted to only 1 contact (inside or outside the household), and (5) teleworking was mandatory.

**Fig. 4:**
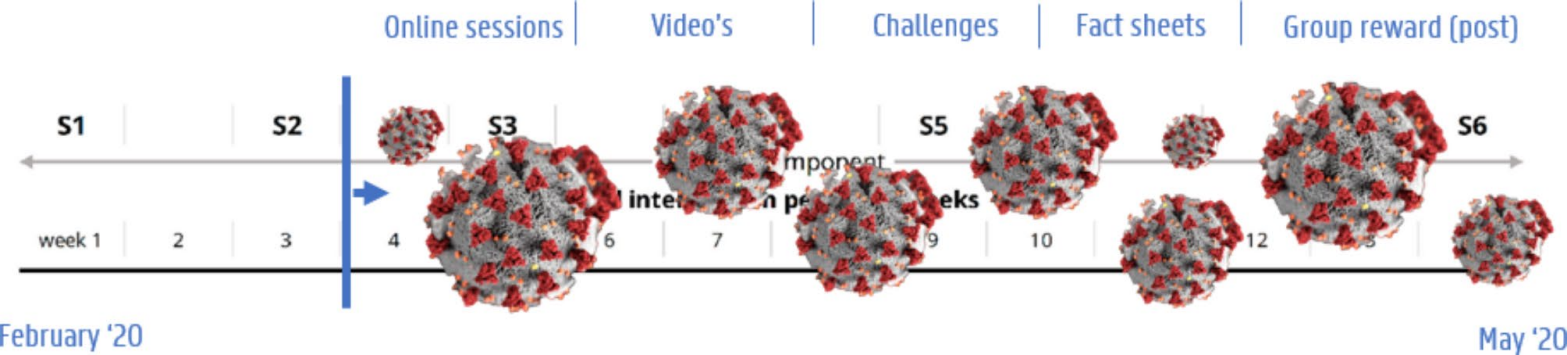
Timeline of the Run Daddy Run intervention under COVID-19 restrictions

The intervention took place between February and May 2020. Due to COVID-19 restrictions, only 2 of the 6 sessions could be implemented in person as planned, between February and March 2020 (see Fig. 4 for a graphical representation). Subsequent sessions were delivered online, from April to May 2020. During this period, “strict” COVID-19 restrictions were applied, similar to the strict measurement described during the follow-up measurements in November 2020. During these strict COVID-19 restrictions, the remaining four face-to-face group sessions were replaced by four online group sessions of ± 30 min. More specifically, a short 30-minutes online version of the practical part of the session was delivered by the facilitators, for each group with fathers and children, on the same dates and times as the session would normally be delivered face-to-face.To replace the face-to-face education part of the sessions, fact sheets were developed and sent by postal mail, covering some of the remaining themes and/or summarizing the most important themes already provided. In between two face-to-face intervention sessions, videos with exercise challenges were sent to fathers to keep them extra motivated. After each session, game materials were sent by postal mail together with a manual and tips on how to use them creatively for exercising together. Last, each time the co-PA group goal was achieved, a congratulation message was posted in the group with the fathers by the facilitators, and a small reward/gadget (e.g. balloon, exercise game) was sent by post to the fathers and children. The online component was implemented as planned throughout the entire intervention period.

### Sample size

The required sample size to evaluate the effect of the intervention was calculated, using the software GPower 3.0.10 [40]. Sample size was calculated for two groups and three time-points of the measurements, with an effect size of f = 0.20, power = 0.80 and alpha = 0.05. A total sample size of 52 father-child dyads was suggested by the power analysis (a priori). Assuming a drop-out of 20% between pre-test and post-test [13], a minimal total sample size of 64 dyads was required in total (i.e. 32 dyads in each group).

### Participants and recruitment

The Run Daddy Run intervention targeted fathers and (one of) their children of the first three years of primary school (i.e. 6–8 years old). For more information on recruitment, see [30]. Inclusion criteria were being the father of a 6–8 years old child; Dutch-speaking; having good health (i.e. no major medical issues or complications); and having a mobile phone with internet [30].

Father-child dyads were recruited through convenience and snowball sampling, in November 2019-January 2020 (see also [30]). More specifically, father-child dyads of both the control and intervention group were recruited through schools, by visiting all 1st grade classes to promote the intervention and by distributing flyers/posters in sports clubs, libraries, and other public places where fathers and or 6–8 years old children could be found. Additionally, fathers were recruited online, through social media such as Facebook and Instagram. Last, registered fathers were also asked to invite other fathers too (e.g. friends or family) to participate in the study. In total, the recruitment phase for the intervention group lasted until it exceeded the available capacity (i.e. 36 father-child dyads). In the control group, no limit was set on the number of participants. In total, 116 families registered for the study (i.e. 64 for the control group and 52 for the intervention group), of which 98 completed baseline measurements (i.e. 63 control group and 35 intervention group) (see Fig. 1 for study design and participant flow).

### Measures

The primary outcomes were co-PA, and total PA of both fathers and their children (i.e. light, moderate and vigorous PA and total volume PA). All the other variables were secondary outcomes (see Table 2 for an overview of all outcomes). During follow-up, only self-report measurements were performed using the online questionnaire (i.e. no accelerometry), due to the strict COVID-19 restrictions applied at that moment which did not allow personal contacts.

**Table 2:** Means, time and interaction effects, and changes in the outcomes from baseline to post-test and follow-up

Outcomes		Pre-test Mean (SE)^a^	Post-test Mean (SE)	Follow-up Mean (SE)	Mean difference between groups^b^ (intervention vs. control) Estimates (SE) [95% CI]		Time x Group (p-value)^c^	
***N***_*primary outcome (co−PA)*_	I	60	45	36	Pre-post	Pre-FU	Pre-post	Pre-FU
	C	31	28	25				
**Main outcomes**								
**Co-physical activity**								
**Total**	I	**12.0 (4.9)**	**38.4 (5.2)**	49.1 (5.5)	**+ 24.2 (7.7)** [9.0; 39.4]	+ 6.4 (8.1) [-9.7; 22.5]	p = 0.002**	p = 0.433
	C	**16.0 (3.7)**	**18.2 (4.1)**	46.7 (4.7)				
**Father-child dyad** **(1 on 1)**	I	**3.9 (2.2)**	**16.7 (2.3)**	**14.0 (2.5)**	**+ 12.9 (3.4)** [6.1; 19.7]	**+ 8.2 (3.6)** [1.0; 15.4]	p < 0.001***	p = 0.027*
	C	**5.2 (1.6)**	**5.1 (1.8)**	**7.1 (2.1)**				
***Standard weekdays*** *(min./day)*	I	**2.1 (2.1)**	**12.7 (2.3)**	11.3 (2.5)	**+ 10.4 (3.9)** [2.7; 18.1]	+ 3.3 (4.1) [-4.9; 11.4]	p = 0.008**	p = 0.431
	C	**3.0 (1.6)**	**3.2 (1.8)**	9.0 (2.1)				
***Weekend days*** *(min./day)*	I	**4.6 (4.6)**	**27.2 (5.1)**	**16.9 (5.4)**	**+ 22.5 (7.7)** [7.2; 37.8]	**+ 18.4 (8.2)** [2.1; 34.7]	p = 0.004**	p = 0.027*
	C	**9.2 (3.6)**	**9.3 (4.0)**	**3.1 (4.6)**				
***Wednesdays*** *(min./day)*	I	3.0 (3.1)	12.3 (3.5)	**18.5 (3.7)**	+ 11.0 (5.8) [-0.5; 22.5]	**+ 13.7 (6.2)** [1.5; 25.9]	p = 0.061	p = 0.028*
	C	5.5 (2.4)	3.9 (2.7)	**7.4 (3.1)**				
**Father-child dyad + other family members**	I	**6.7 (4.3)**	**21.9 (4.7)**	35.1 (5.0)	+ 12.5 (6.9) [-1.1; 26.2]	-0.3 (7.4) [-14.8; 14.2]	p = 0.073	p = 0.967
	C	**11.0 (3.3)**	**13.6 (3.8)**	39.7 (4.2)				
***Standard weekdays*** *(min./day)*	I	4.1 (4.2)	12.4 (4.7)	26.4 (5.0)	+ 5.1 (7.1) [-8.9; 19.2]	+ 3.4 (7.6) [-11.7; 18.4]	p = 0.472	p = 0.659
	C	4.9 (3.3)	8.1 (3.7)	23.9 (4.3)				
***Weekend days*** *(min./day)*	I	**16.1 (8.0)**	**44.5 (8.8)**	48.9 (9.5)	**+ 32.7 (14.1)** [4.7; 60.7]	-2.3 (15.0) [-32.7; 27.5]	p = 0.023*	p = 0.881
	C	**27.1 (6.1)**	**22.8 (7.0)**	62.1 (8.0)				
***Wednesdays*** *(min./day)*	I	6.2 (7.4)	12.8 (8.3)	40.1 (8.9)	-5.11 (13.2) [-31.2; 20.9]	-18.3 (14.1) [-46.0; 9.5]	p = 0.699	p = 0.196
	C	3.0 (5.8)	14.7 (6.5)	55.2 (7.6)				
**PA father**								
***Volume PA*** *(*m*g/day)*	I	**44.1 (2.7)**	**54.8 (2.8)**	n.a.	**+ 6.1 (2.4)** [1.5;10.8]	n.a.	p = 0.010*	n.a.
	C	**46.6 (2.0)**	**51.2 (2.1)**	n.a.				
**LPA** *(*m*g/day)*	I	167.4 (10.6)	184.8 (11.0)	n.a.	+ 3.2 (7.7) [-11.9;18.4]	n.a.	p = 0.676	n.a.
	C	185.6 (8.0)	199.4 (8.2)	n.a.				
**MP** *(*m*g/day)*A	I	**94.6 (10.4)**	**122.3 (10.9)**	n.a.	**+ 17.2 (8.2)** [1.2; 33.3]	n.a.	p = 0.035*	n.a.
	C	**114.9 (7.8)**	**125.4 (8.1)**	n.a.				
**VPA** *(*m*g/day)*	I	6.0 (1.4)	10.2 (1.6)	n.a.	+ 1.9 (1.9) [-1.8; 5.5]	n.a.	p = 0.318	n.a.
	C	3.5 (1.0)	5.9 (1.2)	n.a.				
**PA child**								
**Volume PA** *(*m*g/day)*	I	71.6 (2.7)	73.7 (3.0)	n.a.	-4.3 (3.3) [-10.8;2.1]	n.a.	p = 0.189	n.a.
	C	78.9 (2.0)	85.3 (2.2)	n.a.				
**LPA** *(*m*g/day)*	I	**219.4 (7.7)**	**251.1 (8.5)**	n.a.	**+ 34.6 (8.3)** [18.2;50.9]	n.a.	p < 0.001***	n.a.
	C	**236.5 (5.8)**	**233.6 (6.3)**	n.a.				
**MPA** *(*m*g/day)*	I	**74.0 (4.5)**	**67.8 (4.5)**	n.a.	**-15.2 (5.4)** [-25.8;-4.7]	n.a.	p = 0.005**	n.a.
	C	**80.9 (3.0)**	**90.0 (4.5)**	n.a.				
**VPA** *(*m*g/day)*	I	**7.9 (1.1)**	**7.6 (1.2)**	n.a.	**-4.4 (1.4)** [-7.2;-4.7]	n.a.	p = 0.002**	n.a.
	C	**8.5 (0.8)**	**12.6 (0.9)**	n.a.				
**Secondary outcomes**								
**Body Mass Index**								
**Father** *(kg/m)*	I	25.6 (0.5)	25.7 (0.5)	25.8 (0.5)	+ 0.2 (0.2) [-0.3; 0.6]	+ 0.2 (0.3) [-0.3;0.7]	p = 0.493	p = 0.441
	C	24.9 (0.4)	24.8 (0.4)	24.8 (0.4)				
**Child** *(z-scores)*	I	-0.3 (0.2)	-0.3 (0.2)	0.1 (0.3)	+ 0.2 (0.3) [-0.4;0.7]	+ 0.5 (0.4) [-0.2;1.2]	p = 0.586	p = 0.142
	C	0.0 (0.2)	-0.1 (0.2)	-0.1 (0.2)				
**Sedentary behaviour**								
**Father** *(min./day)*	I	**696.0 (18.9)**	**638.9 (20.3)**	n.a.	**-39.3 (17.2)** [-73.1;-5.6]	n.a.	p = 0.022**	n.a.
	C	**665.2 (14.0)**	**647.4 (14.7)**	n.a.				
**Child** *(min./day)*	I	**555.5 (12.8)**	**539.10 (14.1)**	n.a.	**-40.0 (13.3)** [-65.1;-12.9]	n.a.	p = 0.003**	n.a.
	C	**529.8 (9.7)**	**552.4 (10.4)**	n.a.				
	C	4.6 (0.3)	4.4 (0.3)	4.4 (0.3)				
**Family Health Climate PA (shared perceptions and cognitions on PA)**								
*Score on 14*	I	22.9 (1.0)	24.4 (1.7)	25.9 (1.1)	0.0 (1.1) [-2.3;2.2]	+ 0.8 (1.2) [-1.6;3.3]	p = 0.987	p = 0.492
	C	23.8 (0.7)	25.3 (0.8)	25.9 (0.9)				
**Nurturant fathering scale (quality of father-child relationship)**								
*Score on 45*	I	32.7 (0.7)	33.1 (0.8)	33.9 (0.8)	+ 1.0 (0.8) [-0.7;2.6]	**+ 1.7 (0.9)** [-0.1;3.4]	p = 0.237	p = 0.069
	C	33.9 (0.5)	33.2 (0.6)	33.5 (0.6)				

*p < 0.05, **p < 0.01, ***p < 0.001. In all analyses, there was adjusted for age and BMI of the fathers and children at baseline, and sex (children only)

^a^Means represent the estimated marginal means and their standard errors

^b^Parameter estimates of the fixed effects with their standard errors and 95% confidence intervals

#### Co-physical activity

Co-PA includes all types of PA that involves “active play”, and is jointly performed by the father and the child (e.g. playing soccer together, rough-and-tumble play, cycling). Co-PA was measured using a 7-day recall diary, investigating all physical activities fathers and children performed together in the last seven days [41]. More specifically, fathers were asked to report the start hour of the activity/activities, duration of the activity/activities, with whom they performed these activity/activities, and the kind of activity/activities in this diary (e.g. soccer, cycling), for each day of the week.

Based on this, data were retrieved for co-PA, which consisted of co-PA performed 1 on 1 (father with the participating child) and co-PA conducted with additional family members. Total co-PA was then calculated as the sum of co-PA conducted 1 on 1 + co-PA conducted with additional family members. Furthermore, co-PA was also calculated separately for standard weekdays (which cover all weekdays except Wednesdays), Wednesdays (calculated as a separate variable because it is only a half-day school for the children) and weekend days. All co-PA variables were (calculated and) expressed in average minutes/day.

#### Physical activity and sedentary Behaviour

##### Data collection

Device-based measures of PA and SB data were collected using wrist-worn accelerometers (Axivity AX3, 3-axial) (continuous wave-form data, sampling frequency 100-Hz). Compared to hip-worn accelerometers, wrist-worn accelerometers have better wear time adherence and acceptability [42], and are a valid method to measure physical activity and sedentary behaviour in children and adults [43–45]. The Axivity accelerometers were worn both by the father and the child for 7 consecutive days (24 h protocol) on the non-dominant hand.

##### Data processing

Based on the accelerometer data, participants’ total volume of PA (mean Euclidean Norm Minus One (ENMO, in m*g*) per day), and minutes of light (LPA), moderate (MPA), vigorous (VPA) and sedentary time were assessed during this time period. Upon the return of the Axivity devices, data were extracted using the software OMGUI (Open Movement, Newcastle University, UK) [46]. Data were then processed in R (http://cran.r-project.org) using the open source GGIR package (version 2.1-0) [47, 48]. The signal processing included automatic calibration, detection of sustained abnormally high values, detection of non-wear and calculation of the average magnitude of dynamic acceleration (Euclidean Norm Minus One, ENMO). ENMO was calculated per second by summing the squared acceleration of each of the three accelerometer axes at each time point (i.e. Euclidean Norm) and then subtracting the gravitational component, which is 1 *g* (1 *g*  =  9.81 m/s^2^). The mean ENMO per day (total volume of PA), and minutes in LPA, MPA, VPA and SB per day were then calculated across the monitoring period. To categorize the mean ENMO per minute into the different intensity levels, we used the device-specific prediction equations provided by Hildebrand and colleagues [49, 50] to generate the intensity specific cut-point (adults; SB: 0-45.8 m*g* (milli-gravitational unit), LPA: 45.8–93.2 m*g*, MPA: 93.2-418.3 m*g*, VPA: >418.3 m*g*; children; SB: 0-56.3 m*g*, LPA: 56.3-191.6 m*g*, MPA: 191.6-695.8 m*g*, VPA: >695.8 m*g*). Last, a recently validated nocturnal sleep algorithm included in the software OMGUI was used to detect periods of sleep [51], which was then used to distinguish SB from sleep. Data outside of the 7-day measurement period was excluded. Furthermore, observation days were disregarded completely if weartime during that day was less than 16 h (on the 24 h-protocol), if SB was more than 16 h, and/or if sleep detection was less than 4 h. Non-wear was estimated based on the standard deviation and value range of each axis, calculated for 60 min windows with 15-min moving increments (i.e. the default settings of the GGIR package). If for at least 1 out of the 3 axes the standard deviation was less than 13 m*g* or the value range is less than 50 m*g*, the time window was classified as non-wear. For each participant, data from all valid observation days within the 7-day measurement period was averaged, to obtain and estimation of the participants total volume PA and other objectively measured outcomes.

#### Secondary outcomes: body mass index, PA family health climate and quality of the father-child relationship

Fathers’ Body Mass Index (BMI, in kg/m²) was calculated based on self-reported height and weight through the online questionnaire. BMI z-scores (i.e. a sex- and age adjusted measure of BMI) of children were also calculated based on their weight and height, which were proxy-reported by the father in the questionnaire. The family context on PA (i.e. shared perception and cognitions regarding daily activity behaviours among the family and family members) was surveyed using the validated FHC-PA (i.e. the Family Health Climate Scale for Physical Activity) [52]. Each of the items in this 14-item questionnaire were scored on a 4-point Likert scale (range: “definitely false” to “definitely true”, with a total score ranging from 14 to 56. The father-child relationship was measured with the NFS (i.e. the Nurturant Fathering scale) [53–56]. The items of this 9-item questionnaire were rated on a 5-point Likert scale, with a total score ranging from 9 to 45.

### Data analysis

Descriptive statistics were calculated for the total sample and for the control group and intervention group separately (see Table 3 for the descriptives). Linear mixed models were used to evaluate the intervention effects for all outcomes, taking into account clustering within families to correct for intra-cluster correlation between observations within the father-child dyad. Models were adjusted for age, sex and Body Mass Index (BMI). Interaction effects were reported for pre-post and pre-follow up changes in outcome variables. Additionally, drop-out analyses were conducted using binary logistic regression models to investigate systematic differences between those who dropped out between pre-posttest and pre-follow up test, and those who did not. All statistical analyses were performed using SPSS (v26.0).

**Table 3:** Descriptive statistics of the total sample at baseline

	Fathers			Children		
**N = 98** (35 intervention group, 63 control group)	**Total**	**Intervention**	**Control**	**Total**	**Intervention**	**Control**
**Sex** (% male)	100	100	100	41.8	45.7	40.3
**Age** in yrs (mean, SD)	39.7 (4.5)	39.9 (4.0)	39.6 (4.8)	7.1 (0.9)	7.1 (0.8)	7.0 (0.9)
**BMI** in kg/m² (father) or z-scores (child) *(mean, SD)*	25.1 (3.1)	25.5 (3.1)	24.9 (3.1)	-0.1 (1.3)	-0.3 (1.2)	0.1 (1.3)
**Education level ** *(% high education)*	73.2	79.7	77.9	-	-	-
**Co-PA** in min/day *(mean, SD)*	14.5 (20.4)	11.5 (13.6)	16.0 (23.2)	See father	See father	See father
**Total volume PA** in m*g* /day *(mean, SD)*	45.7 (20.4)	44.6 (21.4)	46.4 (19.8)	75.9 (25.4)	**71.44 (25.89)**	**78.3 (24.8)**
**Total SB** in min/day *(mean, SD)*	673.2 (142.1)	**689.9 (130.9)**	**664.1 (147.2)**	541.3 (98.2)	**558.5 (112.0)**	**531.9 (88.6)**

Note. *Values in bold are p < 0.05*

## Results

### Participant flow

Figure 1 illustrates the flow of the participants throughout the study. In total, 98 father-child dyads provided valid data (i.e. complete data for all the outcomes) at baseline, 90 dyads at post-test and 64 dyads at follow-up (see Fig. 3). From pre to post, drop-out was 8.2% and 34.7% from pre to follow-up. Reasons for drop-out were mostly due to time issues (n = 6), or unknown reasons (i.e. not answering on reason for drop-out, n = 41). For pre-post dropout, attrition analyses showed that father-child dyads including fathers who were older and children with higher BMI z-scores were more likely to drop-out on post-test (see Table 4). For pre- follow-up drop-out, attrition analyses showed that dyads including fathers and children with higher BMI (z-) scores were more likely to drop-out on follow-up. No significant differences were found for the other covariates.

**Table 4:** Results of the (drop-out) attrition analyses for pre-post and pre-follow up dropout

n = 98	Pre-post dropout OR (expB), 95%CI [lower; upper]	Pre-follow up dropout OR (expB), 95%CI [lower, upper]
**Group** (ref. cat. = control group)	0.6 [0.2;2.6]	0.7 [0.3; 1.6]
**BMI father** (kg/m²)	1.2 [0.9;1.5]	1.2 [1.0;1.4]*
**BMI child** (z-scores)	2.1 [1.3;3.5]*	2.0 [1.3;3.1]*
**Age father** (in years	1.1 [1.0;1.3]*	1.0 [0.9;1.3]
**Age child** (in years)	1.4 [0.7;3.0]	1.0 [0.6;1.5]
**Sex child** (ref. cat. = boys)	2.1 [0.5;8.5]	2.3 [1.0;5.6]
**Education level father** (ref. cat. = lower education)	1.7 [0.4;8.7]	1.3 [0.5; 3.4]

*p < 0.05. OR = odds ratio; 95% CI = 95% confidence interval; ref.cat. = reference category. Odds represent the chance on drop-out. Variables in the table represent baseline values

### Descriptive statistics

In total, data of 98 father-child dyads were analyzed at baseline (mean age fathers/male caregivers: 43.79 ± 5.92 years, mean age primary school aged children: 8.19 ± 0.99 years; 50.90% boys). The flow diagram of participants throughout the study can be found in Fig. 3. Descriptive statistics of the sample and variables can be found in Table 3.

### Primary outcomes

#### Co-physical activity

Results for the self-reported co-PA outcomes are shown in Table 2. For total co-PA, a significant Time x Group interaction effect was found from pre- to post-test (+ 24.2 min./day in the intervention vs. control group, p = 0.002). When looking into this effect more closely, the Time x Group effect for co-PA within the father-child dyad was significant for standard weekdays (+ 10.4 min/day, p = 0.008) and weekend days (+ 22.5 min./day, p = 0.004), but not for Wednesdays (p = 0.061) which was, however, significant from pre to follow-up (+ 13.7 min./day, p = 0.028). For co-PA with additional family members, there was found a significant Time x Group effect for weekend days (+ 32.7 min./day, p = 0.023), but not for standard weekdays (p = 0.472) and Wednesdays (p = 0.699). At follow-up, only the effects for co-PA within the father-child dyad (on weekend days) remained significant (+ 18.4 min./day, p = 0.027). For co-PA conducted with additional family members, none of the effects were or remained significant from pre to follow-up (p > 0.05; see Table 2 for a detailed overview of the results).

#### Physical activity

For PA of the father, relative to the control group, the intervention group significantly improved from pre- to post-test for total volume of PA (+ 6.1 m*g*/day in the intervention vs. control group, p = 0.010) and MPA (+ 17.2 min./day, p = 0.035), but no intervention effects were found for LPA and VPA of the father (both p > 0.05). For PA of the child, the intervention group significantly improved from pre- to post-test for LPA (+ 34.6 min./day in the intervention vs. control group, p < 0.001). However, an inverse effect was found for MPA and VPA of the child (MPA: -15.2 min./day in the intervention vs. control group, p = 0.005; VPA: -4.4 min./day in the intervention vs. control group, p = 0.002). Furthermore, no effect was found for total volume of PA (p = 0.189). For a detailed overview of the results, see Table 2.

#### Secondary outcomes: sedentary behaviour, body mass index, PA family health climate and quality of the father-child relationship

Results for the secondary outcomes are shown in Table 2. For SB of the father and SB of the child, there was a significant Time x Group interaction effect from pre- to post-test (p = 0.022 and p = 0.003, respectively). Fathers and children from the intervention group showed a larger decrease in total SB of 39.3 and 40.0 min./day respectively, compared to the control group. For the NFS, the FHC-PA and BMI of the father and the child, no significant interaction effects were found (all p > 0.05).

## Discussion

This study evaluated the effects of a theory-based, co-created intervention aiming to improve co-physical activity (i.e. the father and the child being active together), which was used as a strategy to increase PA in both children and fathers. The ‘Run Daddy Run’ intervention led to an increase in self-reported father-child co-PA, and in objectively measured MPA and total volume PA of the father, and LPA of the child. However, inverse intervention effects were found for MPA and VPA of the child.

Specifically for co-PA, significant intervention effects were found for co-PA conducted *within* the father-child dyad (i.e. conducted 1 on 1) on week- and weekend days (+ 10 min./day and + 23 min./day in intervention vs. control group, respectively), but not on Wednesdays. For co-PA conducted with *additional* family members, a significant increase was found, but only on weekend days (i.e. + 33 min./day in the intervention vs. control group). To follow-up, the effect for co-PA conducted within the father-child dyad on weekend days remained stable, which was 6 months after the intervention without any intervention or contact with the researchers during this period. For co-PA with additional family members on weekend days, the effect was no longer significant at follow-up. A possible reason for the fact that only the effect on co-PA within the father-child dyad remained stable at follow-up, could be because the intervention mainly focused on this specific form of co-PA. A possible explanation for the stability of the co-PA effect on weekend days, is that there was probably more room to engage in co-PA during leisure time, compared to weekdays where there is typically less time for families due to work and school commitments, and (individual) hobbies. However, the follow-up results should generally be considered with caution. The severe COVID-19 restrictions at follow-up, during which outdoor PA was only allowed with a maximum of 4 people, and sports clubs, swimming pools and fitness centers were closed, may have led to a ceiling effect in both the intervention and control group. Consequently, there could be argued that the severe COVID-restrictions on follow-up would have mainly impacted (i.e. increased) co-PA of the entire family, and to a lesser extent on co-PA conducted within the father-child dyad (i.e. co-PA conducted 1 on 1). In the results, this assumption can indeed be confirmed, given the substantial increase in co-PA with additional family members to follow-up in both the intervention and control group, and a less substantial increase or even slight decrease in co-PA performed within the father-child dyad to follow-up. Additionally, the follow-up results indicate that given the opportunity of time, parents can be more active with their children. This might suggest that it is not a lack of motivation that explains the usual low levels of (co-)PA, but other factors (e.g. time constraints), that might hinder the performance of (co-)PA. Future research or data is however needed to confirm this assumption. When comparing these results to the (Australian) HDHK and DADEE programs for fathers and their children, the HDHK and DADEE programs reported increases in co-PA within the father-child dyad ranging from 0.9 to 1.2 days/week at follow-up measurements [38, 57]. In these studies, co-PA was however reported as days/week, where no detailed information on how much and which co-PA was obtained (e.g. minutes per day or with whom it was performed), which makes it difficult to compare these results into more detail.

For objectively measured PA, significant intervention effects were found in the present study for objectively measured MPA of the father (+ 17 min./day in the intervention vs. control group) and total volume PA of the father (+ 6 m*g*/day in the intervention compared to control group). The effect on fathers’ PA are overall positive and clinically relevant, especially as a meta-analysis of Kang et al. (2009) has shown that effects on PA are generally low of PA lifestyle interventions, especially when they involve a combination of age groups like adults and children [58]. When PA data are compared with objective data from the UK Biobank study, a large-scale population-based assessment of PA using objective measurements (i.e. the same devices and similar cut-points as used in the present study), the mean volume of PA in our sample (i.e. 46 m*g* /day) is substantially higher than the mean volume of 7839 male participants of the same age in the UK biobank study (i.e. 31 m*g*/day) [59]. This can be due to the difference in sample size of the UK biobank study versus the Run Daddy Run intervention (i.e. 7800 + participants vs. 98 participants), or to the fact that the Run Daddy Run intervention (i.e. an intervention promoting PA) attracted participants that are slightly more physically active than the general population. When comparing the effects of the Run Daddy Run and the HDHK/DADEE programs, results are comparable [38, 57]. In the latter programs, an increase of 8 to 13 min. MVPA/day was found for fathers of the intervention group at follow-up. This is comparable but slightly less than the increase (in the intervention vs. control group) of 17 min/day MPA and 2 min./day VPA of fathers found at post-test in the present study.

For objectively measured PA in children, an increase in LPA was found in the present study (+ 35 min./day in the intervention vs. control group), but inverse intervention effects were found for MPA and VPA of the child (-15 min./day and − 4 min./day, respectively). A possible explanation for the fact that in the present study only an effect was found for MPA of father and for LPA of children (and a reversed effect for MPA and VPA), could be due to the fact that the (co-)PA promoted in the intervention did not specifically target high-intensity PA for children and adults. In this way, it is possible that children had to lower the intensity level of their PA by being physically active with their father. Although both fathers and children were offered activities of different intensities and difficulty levels during the intervention (i.e. exercises with increasing difficulty and intensity), the fathers but especially the children should be motivated to achieve an optimal intensity level (i.e. MVPA) while performing (co-)PA, and especially outside the intervention context. In this way, their activities performed could achieve a moderate to intense intensity. Nonetheless, multiple studies have shown that any form of exercise (PA), including LPA and MPA, already has positive effects on health, both in adults and in children [60, 61].

Regarding the secondary outcomes, significant decreases in fathers’ and children’s objectively measured SB were found, which is again comparable to the results of the Australian HDHK/DADEE programs [38, 57]. This is an important finding, as PA interventions are often not able to reduce SB [62]. Furthermore, the effects found were greater than the current estimates from a recent meta-analysis and systematic review on existing SB interventions in both children and adults [63, 64]. The Run Daddy Run intervention is therefore one of few lifestyle interventions that is able to positively change PA and SB at the same time, in both adults and children. However, in the present study, the decrease in SB was not fully covered by the increase in PA (in both fathers and children). This can possibly be explained by non-wear time of the accelerometer. Indeed, the non-wear time from pre- to post-test differed significantly between the intervention group and the control group, with the intervention group wearing the device approximately 30 min less, compared to the control group. To take this into account, the analyses were repeated for objectively measured PA and SB, of both the fathers and children, with wear-time included as a covariate, but this did not significantly change any of the intervention effects. The effects slightly attenuated for sedentary behavior (i.e. a smaller decrease in SB of both fathers and children), which confirms that non-wear time only had a small influence.

Last, the intervention did not significantly change the weight status of fathers and children. These findings are in line with the Australian DADEE study, which also did not find an effect on BMI (of both fathers and children) at follow-up [38]. In contrast, results of the HDHK study did show an effect on BMI, with a decrease of 7.6 kg in the intervention group compared to the control group. Importantly, these Australian studies recruited and targeted specifically overweight and obese fathers (i.e. mean BMI of the fathers was 33.2 kg/m² in the HDHK RCT and 27 kg/m² in the DADEE program), which was not the case in our study were the general population (independent of weight status) was invited to participate (i.e. mean BMI of fathers in the Run Daddy Run intervention was 25.1 kg/m²). Furthermore, it is not unusual that no effect on weight status is found immediately after the intervention. A longer time period is probably needed to see effects on this outcome. Nevertheless, the main aim of the Run Daddy Run intervention was the prevention of overweight and obesity by increasing co-PA, rather than weight loss. In children, a stable effect was therefore the most desirable effect, since they already had a normal weight at baseline. Another explanation of the non-significant effect on weight status might be due to the measure that was used for weight status (i.e. BMI for adults and BMI z-scores for children) These are the most commonly used measures in research to classify overweight and obesity, as it are unobtrusive measures that are easy to obtain because they are often based on self-reported or objectively measured weight and height, and they correlate high with body adiposity [23]. However, it should be kept in mind that these are still proxy measures of body composition and can only provide a rough estimate of adipose tissue [24]. Complementing these measurements by other measurements of adiposity, for example, waist circumference (which is a good measurement for abdominal fat in both adults and children), skinfold thickness measurements (which is a good measure for subcutaneous fat) or waist-to-height ratio is therefore recommended for future research [25, 26]. Last, the lack of a significant intervention effect can also be due to the fact that BMI was not objectively measured, but obtained by self-report (fathers) and proxy-report (children). Although self-report instruments can provide accurate and valid assessments of health-related behaviors and outcomes with good participation compliance, they are still subject to social desirability and response bias [27].

Last, also no significant changes were observed for the father-child relationship and the family health climate regarding PA (all p > 0.05). This could possibly be due to the fact that because of COVID-19 restrictions, not all sessions were implemented as planned. More specifically, only two out of six sessions could be implemented face-to-face as planned, the remaining sessions were delivered online for the fathers and children (i.e. four active face-to-face sessions of about 30 min), replacing the remaining face-to-face sessions as much as possible. In between two sessions, videos with exercise challenges were sent to fathers to keep them motivated. After each session, game materials were sent by postal mail together with a manual and tips on how to use them creatively for exercising together. To replace the education part of the sessions, fact sheets were developed and sent by postal mail, covering some of the remaining themes and/or summarizing the most important themes already provided. However, it is possible that the fathers did not read these fact sheets which consequently can explain why no significant changes were observed for the father-child relationship and the family health climate.

Taken together, positive intervention effects of the Run Daddy Run intervention were still found with a total face-to-face contact time of 360 min (instead of the originally planned 720 min). Besides the positive effects, the online alternatives were also positively perceived by the participants. More specifically, in the process evaluation questionnaire, participants of the Run Daddy Run intervention indicated that the researchers provided useful and stimulating alternatives for the intervention during COVID-19. Based on these findings, we might assume that a limited amount of face-to-face contact, in combination with an online component throughout the entire intervention period, may be sufficient to induce and motivate fathers and children towards behavior change. This is an interesting finding, as eHealth interventions have indeed emerged as promising and effective for improving physical activity, mainly because of their ability to provide efficient, interactive and tailored information and content to the user [30]. Moreover, such an online component is more cost- and time effective than implementing several face-to-face sessions, as it puts minimal burden on the researchers and facilitators of the program [31]. Yet, and this was suggested in a meta-analysis of Young et al. [32], face-to-face contact is still very important for fathers as a target group, especially as it appears to enhance group dynamics and motivation. Thus, it can be suggested for future interventions aiming to improve lifestyle behaviors in fathers and children, to provide at least two to three face-to-face contact moments per month in group, providing reminders between the sessions, and combine this with an online component that stimulates motivation for behavior change both individually and for the entire group, throughout the entire intervention period.

Strengths of the present study include the theory-based approach which was combined with co-creation, taking into account the needs and the preferences of fathers who are difficult to engage in these kind of programs; the high program attendance and low drop-out and strong retention rates, supporting the acceptability of this study; the use of objective PA data where cut-offs from the same authors (i.e. Hildebrand et al. [49, 50]) were used to classify the PA intensities of the adults and children; the use of detailed and precise information on the main outcome variable co-PA; and the inclusion of follow-up measurements (i.e. 6 months after the posttest measurements). However, there were also some study limitations. A first limitation was the non-randomized controlled design in which the participants were recruited in sequence (i.e. first the control group, then the intervention group). This may have caused an allocation bias, where systematic differences arise from in how participants are assigned to the intervention vs. control group. Appropriate randomizing is suggested to prevent this bias, i.e. by conducting a randomized controlled trial (RCT). Second, a self-report measure (i.e. 7-day recall diary) was used to measure co-PA. Although diary-based measurements can lead to an accurate and valid assessment of PA [41], this method can still be subject to social desirability. Objective measurements, using Bluetooth proximity sensing for measuring co-PA is suggested for future research [65]. A further limitation is that there were baseline differences between the control group and intervention group in PA (total volume) of the children, and SB of both the children and the fathers, with fathers and children of the intervention group being significantly less active and more sedentary than the control group. This could possibly be due to the fact that our intervention group had a slightly higher BMI than the control group, although this difference was not significant. On the other hand, the time of measurement may have played a role in this, or the difference in sample size. Although the latter was not a consequence of the design, but rather a practical limitation (i.e. independent of the participants), it does result in less statistical power. In future studies, a limit can be set on the number of participants in the control group, in accordance with the allowed number of participants in the intervention group.

Future studies could adapt the (co-PA) activities so that they target and optimally stimulate MVPA in both children and adults. Efforts should also be made to stimulate (co-)PA on days on which father and children have less time and are busier, for example on weekdays. Furthermore, it could be examined whether this intervention is also suitable for a diverse range of families. For example, it could be explored whether this intervention is also suitable for an overweight/obese population, and/or lower educated or ethnic minority groups. If not, new co-creation sessions can be organized so that appropriate and required adaptations can be made. Last, future research is needed to confirm the scalability and practical feasibility of the program in a community trial. However, this programme has currently been adopted by Families Sports Flanders in which the intervention will be implemented on a larger scale in East-Flanders (Belgium), where its effects can be evaluated in a community setting too.

## Conclusion

This study has shown that the Run Daddy Run intervention -as applied during the COVID-19 restrictions at that time- was effective in improving co-PA, objectively measured MPA and total volume PA of the father, LPA of the child, and SB levels of both fathers and their children. Efforts should however be made to target PA of higher intensities in the intervention, especially children’s MPA and VPA. These significant and clinically relevant findings, which sustained over time, show that involving fathers might be a novel approach to improve health behaviours in children. Future research is however needed to confirm the scalability and practical feasibility of the program, and to explore whether this intervention is suitable for a diverse range of families. Last, replicating these findings in a randomized controlled trial (RCT) is also recommended.

## Data Availability

The datasets used and analyzed during the current study, and the full trial protocol, are available from the corresponding author on reasonable request.

## Abbreviations

PA: Physical activity.
LPA: Light physical activity.
MPA: Moderate physical activity.
VPA: Vigorous physical activity.
MVPA: Moderate-to-vigorous physical activity.
SB: Sedentary behaviour.
m*g*: milli-gravitational unit.
ENMO: Euclidean Norm Minus One.
Co-PA: Co-physical activity.
FMS: Fundamental Movement Skills.
